# Grouping strategies in numerosity perception between intrinsic and extrinsic grouping cues

**DOI:** 10.1038/s41598-021-96944-x

**Published:** 2021-09-02

**Authors:** Yun Pan, Huanyu Yang, Mengmeng Li, Jian Zhang, Lihua Cui

**Affiliations:** 1 Key Laboratory of Basic Psychological and Cognitive Neuroscience, School of Psychology, Guizhou Normal University, Guiyang, China; 2 Education School, Yunnan University of Business Management, Kunming, China

**Keywords:** Psychology, Human behaviour

## Abstract

The number of items in an array can be quickly and accurately estimated by dividing the array into subgroups, in a strategy termed “groupitizing.” For example, when memorizing a telephone number, it is better to do so by divide the number into several segments. Different forms of visual grouping can affect the precision of the enumeration of a large set of items. Previous studies have found that when groupitizing, enumeration precision is improved by grouping arrays using visual proximity and color similarity. Based on Gestalt theory, Palmer (Cognit Psychol 24:436, 1992) divided perceptual grouping into intrinsic (e.g., proximity, similarity) and extrinsic (e.g., connectedness, common region) principles. Studies have investigated groupitizing effects on intrinsic grouping. However, to the best of our knowledge, no study has explored groupitizing effects for extrinsic grouping cues. Therefore, this study explored whether extrinsic grouping cues differed from intrinsic grouping cues for groupitizing effects in numerosity perception. The results showed that both extrinsic and intrinsic grouping cues improved enumeration precision. However, extrinsic grouping was more accurate in terms of the sensory precision of the numerosity perception.

## Introduction

Numerosity perception is a quantitative attribute of entities that is an important dimension of nature. For example, one tree produces more fruits than another, and there are more sheep in one territory than in another. In the process of understanding and adapting to nature, humans and animals have gradually evolved in their ability to perceive numerosity^1–3^.

Three strategies are usually used in numerosity perception: subitizing, counting, and estimation^4,5^. For a small number of items (usually less than 4), humans can quickly and accurately determine the number of items in the clusters. This is called “subitizing,” which indicates that the items can be understood immediately, without thinking^4,6,7^. With an increasing number of items (greater than 4), the time required to determine the number of items also increases correspondingly while requiring the coordination of many visual and spatial operations, as the observer determines the number of objects by counting^8,9^.When the number of items in clusters is large and cannot be counted in a very short time (for example, the presentation time of the stimulus is short), numerosity perception may be inaccurate, pursuant to Weber's Law, the approximate number system (ANS) can be relied upon^10^. People thus use estimation strategy to determine the approximate number of objects^10–12^.

Beyond subitizing, counting and estimation, recent studies have found that arrays visually divided into sub-groups can be enumerated faster and more accurately than ungrouped arrays; this is called “groupitizing”^4,13–17^. An increasing number of studies have begun to explore the mechanisms of groupitizing. For example, Starkey and McCandliss (2014) found that groupitizing is positively correlated with the arithmetic ability of children and adults, indicating that the grouping ability may reflect the arithmetic strategies^13^. Moscoso et al. (2020) suggested that groupitizing is a process based on attention, which depends on the subitizing system, and mathematical ability is correlated with groupitizing^15^. Ciccione and Dehaene (2020) indicated that in groupitizing, subjects use mental multiplication and mental addition to increase the speed and accuracy of enumeration^4^. Wege et al. (2021) explained that the numerical information needed for a mental calculation is extracted from grouped arrays, and suggested that the parallel subitizing of dots and groups in grouped arrays may represent the enumeration processes necessary for groupitizing via mental multiplication^18^.

The groupitizing effect must be based on visual grouping. Research into visual grouping processes and perceptual organization developed against the background of Gestalt theory approximately one hundred years ago^19–21^. Wertheimer (1923) proposed the main principles of perceptual grouping, which specified which regions of images constituted objects or perceptual units, such as similarity, proximity, symmetry, good continuity^21^. Based on Gestalt theory, Palmer made an important distinction between intrinsic and extrinsic grouping principles. Like most classical Gestalt principles, intrinsic principles are based on the inherent relationships among attributes of grouped elements (e.g., color, shape, size, position). In contrast, the extrinsic principles are based on relationships among elements and other extrinsic elements that induce them to group (e.g., connectedness or common region)^22–25^.

Previous studies of groupitizing only involved intrinsic grouping cues (color similarity^4,14^ and proximity^4,13–15^). To date, no research has explored numerosity perception with extrinsic grouping cues. Thus, this study explored whether extrinsic grouping cues are different from intrinsic grouping cues in numerosity perception. Previous studies have found that extrinsic grouping cues have advantages over intrinsic grouping cues^22,26–31^. Luna et al. suggested that observers respond more quickly to extrinsic cues than to other grouping cues^22,32^. Quinn and Bhatt reported that young infants (3–4 months old) are sensitive to extrinsic cues, especially common region and connectedness^22,28^. Therefore, we hypothesized that extrinsic grouping cues would be advantageous in numerosity perception.

In addition, vision research has revealed that shape is crucial for object recognition^33–36^. Without other visual information, it is easy for humans to use shapes to identify objects. Human adults and children prefer to classify new objects according to their shape, given conflicting color and texture cues^33,36,37^. Accordingly, in this study, a shape similarity cue was added to the intrinsic grouping cues to verify whether the shape similarity grouping cues have different effects than other intrinsic grouping cues (i.e., color similarity and proximity).

## Methods

### Participants

Fifty-three freshman college students (mean age = 19 years, standard deviation = 2.4, range = 18–22) with normal (or corrected-to-normal) vision, and no color blindness were selected. We replicated the experiment in three groups of participants with low, medium, and high levels of math knowledge (for a similar approach, see Dehaene et al., 2020)^4^. At the highest level, we tested 16 science students majoring in mathematics, all of whom had scored over 120 points on China’s mathematics college entrance examination in 2020. For the medium level, we tested 18 humanities students with math scores between 60 and 90 in the college entrance examination (the mathematics component of the college entrance examination for science is more difficult than for humanities. The maximum score for mathematics is 150). We tested 19 students in the low-level group. They were sports students who had never taken university-level exams in mathematics or related disciplines. The third group was less familiar with mathematics as they had not been taught mathematics for at least one year.

### Materials and procedure

**Figure 1:**
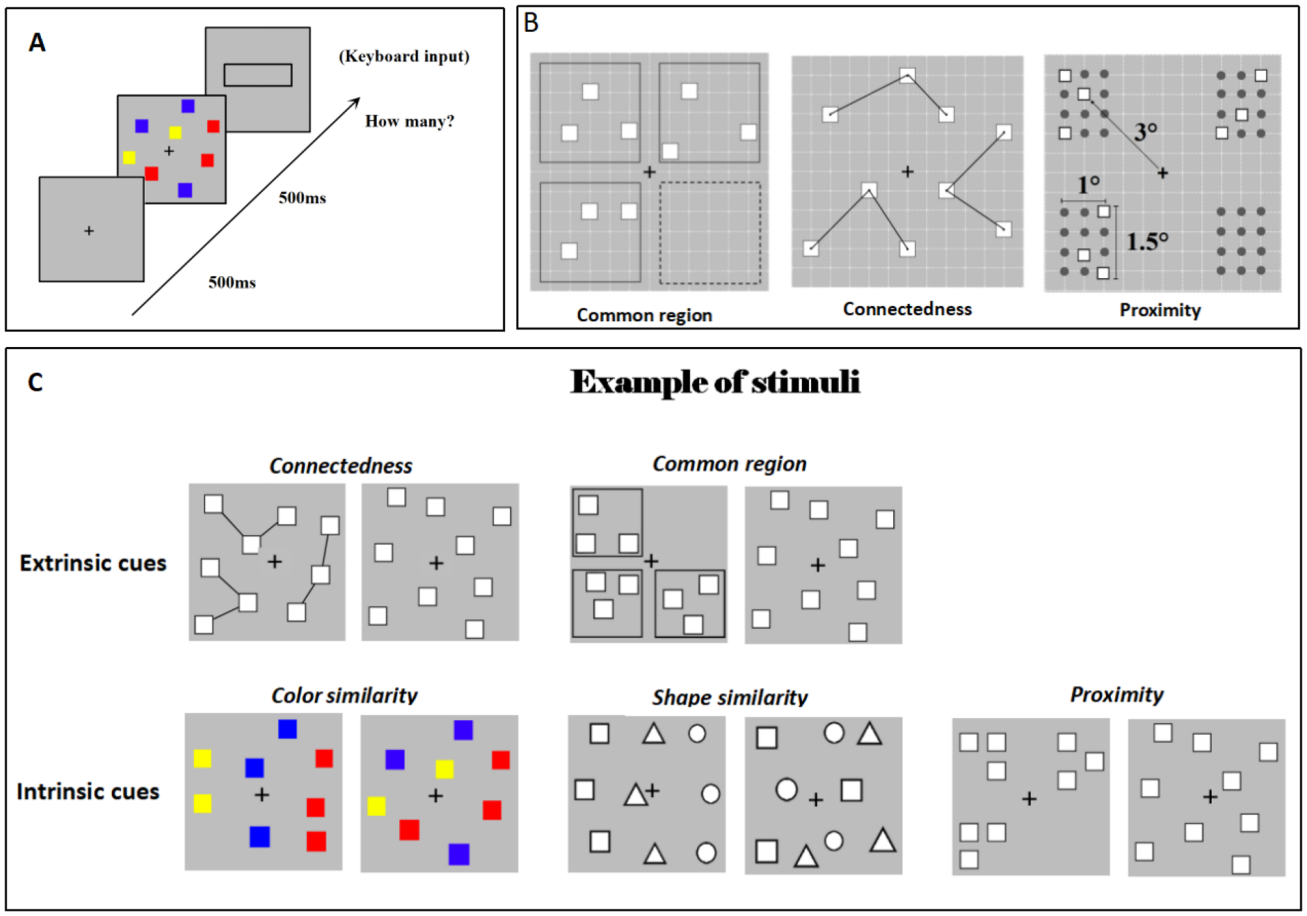
Illustration of the stimuli and procedure. (**A**) Example of the time course of the experiment. (**B**) Illustration of how stimulus position was defined in the grouping conditions. (**C**) Example of stimuli. Stimuli are not depicted to scale. Stimuli figures can be found as supplemental figures on the OSF page.

Stimuli were presented using E-Prime 2.0. Participants sat in a quiet and dimly light room, 60 cm from a screen monitor (60 Hz). At the beginning of each trial, a fixation point was presented in the center of the screen and remained on the screen throughout the experiment. After 500 ms, a stimulus was displayed for 500 ms, followed by a screen with an input box. Participants estimated the number of stimuli present and entered the estimated result into the input box as quickly and accurately as possible using a numeric keypad (Fig. 1A). Response time was measured from stimulus offset to when the input box was presented. Each condition was tested in separate blocks, and participants were never explicitly informed of the grouping cues.

### Stimuli

All stimuli were distributed in a 6° × 6° square grid, which consisted of 144 small squares, where each square was 0.4° × 0.4°, and the array was placed at the intersection of the grids, so that each item had 121 possible positions (Fig. 1B). We tested all numerosities between 5 and 17; there were 13 different numerosities. In the grouping conditions, each numerosity was divided into 2–4 subgroups, and each subgroup contained between 2 and 6 items, configurations were as following: 2, 2, 1; 3, 3; 3, 3, 1; 2, 2, 2, 2; 3, 3, 3; 3, 3, 3, 1; 3, 3, 3, 2; 3, 3, 3, 3; 5, 5, 3; 4, 4, 3, 3; 4, 4, 4, 3; 4, 4, 4, 4; 5, 4, 4, 4. The participants were clearly informed of the range of the numerosity. Each participant completed 390 trials in total, with each numerosity presented in 30 times. “Stimuli Details” files on the OSF page.

#### Extrinsic cues

Extrinsic cues included connectedness and common region.

#### Connectedness

In the connectedness conditions, the stimuli were sets of white squares (0.4° × 0.4°) with black borders randomly distributed in the grid. The squares within subgroups were connected by a black line, with the connection at the center of the square. In the no-grouping condition, there was no black line connection, and each item was randomly distributed in the large grid (Fig. 1B).

#### Common region

In the common region conditions, stimuli were also setting of white squares (0.4° × 0.4°) with black borders. The grid was divided into four quadrants, and the squares of each subgroup were randomly distributed inside the small square boxes (2.5° × 2.5°) in the four quadrants. For example, Fig. 1B is a 3, 3, 3 group with only three subgroups, so there are only three boxes. In the no-grouping condition, there were no small square boxes, and each item was randomly distributed in a large grid (Fig. 1B).

#### Intrinsic cues

Intrinsic cues included color similarity, shape similarity, and proximity.

#### Color similarity

The color similarity conditions were the same as those used by Anobile et al. (2020)^14^. Individual items (0.4° × 0.4°) could be red, blue, yellow, or green, (RGB: 255 0 0; 0 0 255; 255 255 0; 0 255 0, respectively). Colors were arranged from left to right, so that similar colors appeared in a vertical column (see Fig. 1C for a 3, 3, 3 group), where squares were first randomly arranged. The first three squares were colored red (from the left to right), the next three blue, and the remaining three yellow (colors were randomly selected for each group). In the no-grouping condition, positions of the squares were arranged with the same logic, but the colors were randomly assigned.

#### Shape similarity

The shape similarity condition was similar to the color similarity condition. The only difference was that four shapes replaced the four colors: square, circle, triangle, and diamond; all shapes were 0.4° × 0.4°, and white with black borders (Fig. 1C).

#### Proximity

The proximity conditions were the same as those used by Anobile et al. (2020)^14^. Stimuli were arranged into four possible groups of 12 possible positions. Each group (spanning a maximum area of 4° × 2°) was located in the same quadrant and centered at 5° from the central fixation point. Each group was first randomly assigned to one quadrant (between 1 and 4); then, the individual item positions were randomly selected between one of the 12 positions in the selected quadrant. Within each quadrant, the maximum center-to-center distance between each element was 4°, and the minimum was 1°. In the no-grouping condition, each item was randomly distributed in the large grid (Fig. 1C).

### Data analysis

To statistically test differences across conditions. We adopted repeated-measures ANOVA, which included the grouping condition (2 levels for grouping and no-grouping), grouping cue (5 levels for connectedness, common region, color similarity, shape similarity, proximity), and numerosity (13 levels, from N5 to N17) as within-subjects factors. Math knowledge (high, medium, and low) was a between-subjects factor.

The median reaction times (RTs) for correct answers were computed for each subject. We excluded trials with RTs more than three standard deviations from the average RTs. Precision was measured by the coefficient of variation (CV), which is a dimensionless precision index that allows cross-numerical comparison of average performance.

1 $$ {\text{CV}} = \frac{i}{Ni} $$

*N*_*i*_ is the analyzed numerosity, and *i* is the standard deviation of the responses to numerosity *i*.

Data were analyzed by repeated measures ANOVA and *t*-tests, using JASP statistical package version 0.14.1.0 (https://jasp-stats.org) and IBM SPSS Statistics version 19 (http://www.spss.com/). In addition, we used Bayesian ANOVA inference for additional analysis, because quantifying evidence in favor of both difference and equality was crucial for testing our hypotheses (Wagenmakers et al. 2018)^38^. We reported the Bayes factors in favor of the alternative (BF_10_). A BF_10_ larger than 1 indicated evidence supporting the alternative hypothesis, and a BF_10_ less than 1 indicated evidence for the null hypothesis. We applied Bonferroni corrections to all post hoc analyses to correct for multiple comparisons.

(The full raw data from this experiment are available on our OSF page. We thank an anonymous reviewer for this suggestion).

### Statement

All coauthors agreed with the contents of the manuscript. The study with human subjects was conducted in accordance with the Declaration of Helsinki. This study was approved by the School of Psychology Ethics Committee at Guizhou Normal University. All participants signed informed consent forms prior to the experiment. All methods were carried out in accordance with relevant guidelines and regulations.

## Results

As in several previous studies^14,15^, we also investigated grouping effects on RT and sensory precision (as indexed by the coefficient of variation CV) (Eq. 1). CV is a classical psychophysical parameter; in numerosity perception, this parameter reflects the sensory noise associated with the estimation process: the higher the CV value, the more sensory noise, and thus the less precise the estimates.

Tables 1 and 2 shows the main effect and interaction of ANOVA for RT (Table 1) and CV (Table 2).

**Table 1:** Mixed-model repeated-measures ANOVA for RT.

Effect	*df*	*F*	*p*	BF_**10**_	Partial *η*^2^
Numerosity	12.39	289.345	< 0.001***	> 100	0.989
Grouping cue	4.47	15.526	< 0.001***	> 100	0.569
Grouping condition	1.50	13.001	< 0.001***	> 100	0.995
Numerosity × Grouping cue	48.3	4.798	0.028*	> 100	0.987
Numerosity × Grouping condition	12.39	55.306	< 0.001***	> 100	0.944
Grouping cue × Grouping condition	4.47	18.451	< 0.001***	> 100	0.611
Numerosity × Grouping cue × Grouping condition	48.3	27.912	0.009**	> 100	0.998
Math knowledge	2.52	4.798	0.012*	> 100	0.016
Math knowledge × Numerosity	12.40	1.924	0.016*	> 100	0.366
Math knowledge × Grouping cue	4.48	2.807	0.069	> 100	0.190
Math knowledge × Grouping condition	2.50	1.496	0.004**	> 100	0.056
Math knowledge × Numerosity × Grouping cue	48.4	1.795	0.190	0.000	0.956
Math knowledge × Numerosity × Grouping condition	12.40	1.434	0.088	0.000	0.301
Math knowledge × Grouping cue × Grouping condition	4.48	0.536	0.827	0.000	0.43
Math knowledge × Numerosity × Grouping condition × Grouping cue	48.4	2.221	0.112	0.000	0.964

**Table 2:** Mixed-model repeated-measures ANOVA for CV.

Effect	*df*	*F*	*p*	BF_**10**_	Partial *η*^2^
Numerosity	12.39	96.834	< 0.001***	> 100	0.968
Grouping cue	4.47	2.894	0.034*	> 100	0.195
Grouping condition	1.50	2.807	< 0.001***	> 100	0.558
Numerosity × Grouping cue	48.3	4.798	0.11	> 100	0.987
Numerosity × Grouping condition	12.39	7.395	< 0.001***	> 100	0.695
Grouping cue × Grouping condition	4.47	1.495	0.219	0.000	0.113
Numerosity × Grouping cue × Grouping condition	48.3	2.197	0.285	0.000	0.972
Math knowledge	2.52	4.798	0.012*	> 100	0.016
Math knowledge × Numerosity	12.40	1.279	0.206	> 100	0.277
Math knowledge × Grouping cue	4.48	2.807	0.008**	0.11	0.190
Math knowledge × Grouping condition	2.50	1.496	0.234	1.50	0.056
Math knowledge × Numerosity × Grouping cue	48.4	1.009	0.552	0.000	0.924
Math knowledge × Numerosity × Grouping condition	12.40	0.697	0.840	0.000	0.173
Math knowledge × Grouping cue × Grouping condition	4.48	1.267	0.27	0.000	0.096
Math knowledge × Numerosity × Grouping condition × Grouping cue	48.4	1.034	0.533	0.000	0.925

### Groupitizing and grouping cues

**Figure 2:**
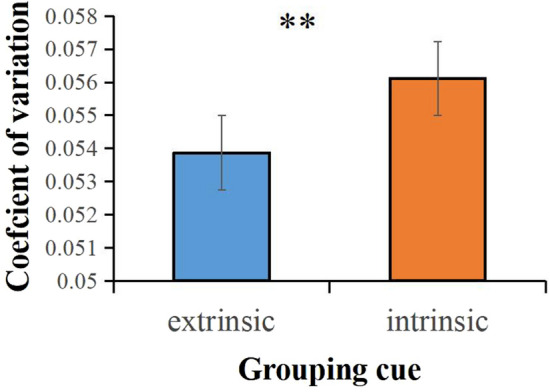
Results of extrinsic and intrinsic grouping cues. CV for extrinsic and intrinsic grouping cues by group condition. ***p < 0.001; **p < 0.01; *p < 0.05.

We compared the numerosity perception of extrinsic grouping cues (connectedness, common region) and intrinsic grouping cues (color similarity, shape similarity, proximity), average across numerosities and conditions. A *t*-test revealed strong statistical evidence for the differences between the extrinsic and intrinsic grouping cues for CV (*p* < 0.01). As shown in Fig. 2, the CV for extrinsic grouping cues was lower than that for intrinsic grouping cues, indicating that the sensory precision of extrinsic grouping cues was more accurate (less sensory noise) than that of intrinsic grouping cues (Fig. 2). But the extrinsic and intrinsic grouping cues differences for RTs were not significant.

**Figure 3:**
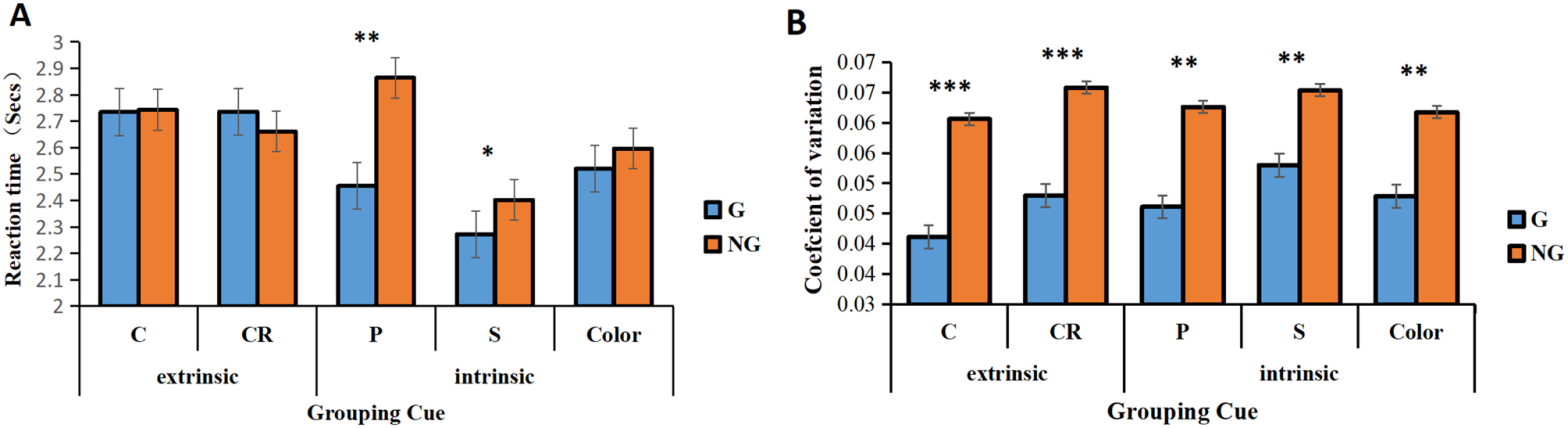
Grouping cue effect. RTs (**A**) and CV (**B**) for different grouping cues by group condition (G for grouping condition, NG for no-grouping condition; C for connectedness, CR for common region, P for proximity, S for shape, Color for color). ***p < 0.001; **p < 0.01; *p < 0.05.

The ANOVA of RTs revealed a significant main effect of the grouping condition, *F* (1, 51) = 13.001, *p* < 0.001***, BF_10_ > 100, the grouping conditions reacted faster than that in no-grouping conditions. And the main effect of grouping cue was also significant, *F* (4, 47) = 15.526, *p* < 0.001***, BF_10_ > 100. The interaction between grouping cue and grouping condition was significant, *F* (4, 47) = 18.451, *p* < 0.001***, BF_10_ > 100. We performed a simple effects analysis to further test the differences in grouping conditions at different grouping cues. The result can be seen from Fig. 3, for extrinsic grouping cues, there was no significant difference in RT between grouping and no-grouping conditions. But for intrinsic grouping cues, proximity and shape similarity grouping cues had a better grouping effect (the reaction was significantly faster in the grouping condition than in the no-grouping condition) (Fig. 3).

The ANOVA of CV revealed a significant main effect of grouping conditions, *F* (1, 50) = 2.807, *p* < 0.001***, BF_10_ > 100, and the grouping conditions had lower CV than no-grouping conditions, which means that grouping condition had less sensory noise and more accuracy. The ANOVA of CV also revealed a significant main effect of grouping cue *F* (4, 47) = 2.894, *p* = 0.034*, BF_10_ > 100; but its interaction with the grouping condition was not significant.

### Groupitizing and specific numerosity

**Figure 4:**
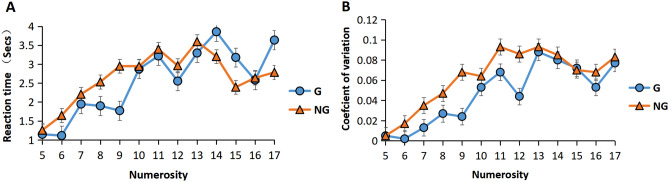
Specific numerosities. RTs (**A**) and CV (**B**) for different numerosities across group conditions.

ANOVA of RTs revealed a significant main effect of numerosity *F* (12, 39) = 289.35, *p* < 0.001***, BF_10_ > 100. From Fig. 4A, it is evident that RT increased linearly with numerosity, small numerosities reacted significantly faster than large numerosities, and the interaction with the grouping condition was also significant, *F* (12, 39) = 55.306, *p* < 0.001***, BF_10_ > 100. We performed a simple effects analysis to further test the differences in grouping conditions at different numerosities and examined how RTs varied with numerosity in each condition. The results showed that, in the grouping condition, numerosities 6, 9, 12, and 16 had faster RTs than adjacent numbers. In contrast, numbers 7, 11, 13, 17 had slower RTs than their neighbors (Fig. 4A).

ANOVA of CV revealed a significant main effect of numerosity, *F* (12, 39) = 96.83, *p* < 0.001***, BF_10_ > 100. From Fig. 4B, it is evident that CV increased linearly with the numerosity and small numerosities were more accurate than large numerosities. The interaction with the grouping condition was also significant, *F* (12,39) = 7.395, *p* < 0.001**, BF_10_ > 100. Similar to RT, in the grouping condition, numerosities 6, 9, 12, and 16 had lower CV than their neighbors (Fig. 4B). The results were consistent with previous research^4,14,15^.

**Figure 5:**
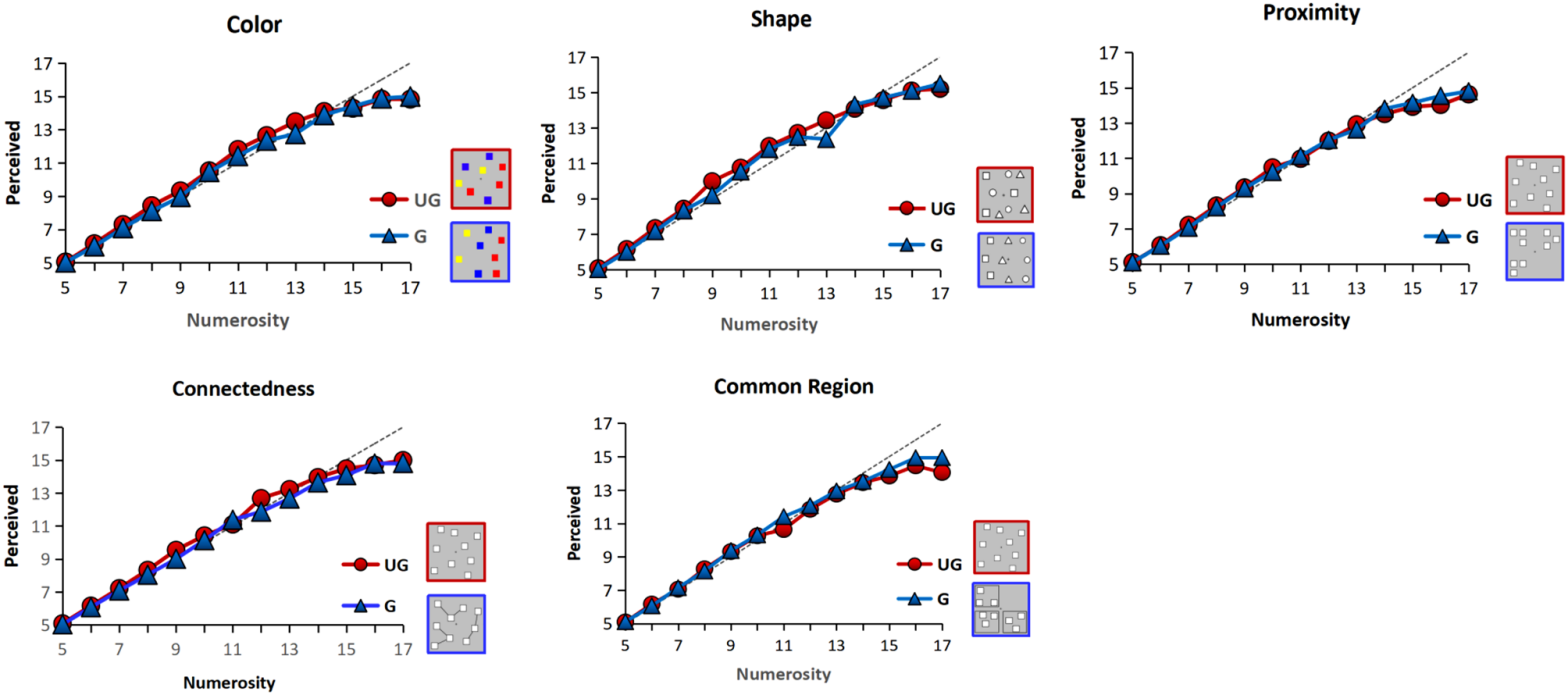
Perceived numerosity. Average perceived numerosity for all grouping cues and tasks, averaged across participants.

Moreover, we found that large numerosities were underestimated for each grouping cue (Fig. 5). In Fig. 5, the dotted line represents the accurate perception of numerosities. Above the dotted line indicates overestimation, while below the dotted line indicates underestimation. From Fig. 5, we can see that under all grouping cues, the subjects began to be underestimated from the value of 13, consistent with the results of previous studies^14^.

### Influence of math knowledge

**Figure 6:**
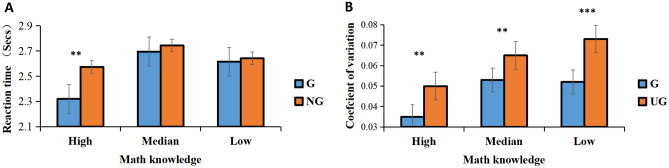
Results for math knowledge. RTs (**A**) and CV (**B**) in groups with high, medium, and low levels of math knowledge by group condition. ***p < 0.001; **p < 0.01; *p < 0.05.

We compared grouping effects for subjects with high, medium, and low levels of math knowledge. ANOVA of RTs revealed a significant main effect of math knowledge, *F* (2, 52) = 4.798, *p* = 0.012, BF_10_ > 100. The subjects in the high math knowledge group had faster RTs, and its interaction with the grouping condition was also significant, *F* (2, 50) = 1.496, *p* = 0.004, BF_10_ > 100. We performed a simple effects analysis to further test the differences in grouping conditions at different levels of math knowledge. We found that the grouping effect was significant only in groups with high math knowledge (see Fig. 6A).

ANOVA of CV also revealed a significant main effect of math knowledge, *F* (2, 52) = 4.798, *p* = 0.012*,* BF_10_ > 100, the subjects in the high math knowledge group had lower CV; but its interaction with the grouping condition was not significant (Fig. 6B).

## Discussion

Our results showed that when items were divided into several subgroups, this benefited perception of the numerosity. Furthermore, according to Gestalt theory, perceptual grouping can be divided into extrinsic and intrinsic grouping cues^23–25^. Accordingly, this study explored whether different grouping cues had different influences on groupitizing. The results showed that the sensory precision of extrinsic grouping cues was more accurate than that of intrinsic grouping cues, and the grouping effect was stronger.

The RT for extrinsic grouping cues and intrinsic grouping cues was not significant, inconsistent with Luna et al. (2016), who found that extrinsic grouping cues, especially common regions, were associated with faster RTs than other grouping cues^22,32^. In addition, Quinn and Bhatt (2015) also found that early infants (4–6 months) were more sensitive to extrinsic grouping cues^26,28^. Although the RTs for extrinsic grouping cues and intrinsic grouping cues were not significant, the sensory precision of extrinsic grouping cues were more accurate than that of intrinsic grouping cues (Fig. 3). This may indicate that extrinsic grouping cues have a strong advantage of groupitizing. Compared with intrinsic grouping cues, the addition of connecting lines or closing lines led to more visual interference. It also required additional cognitive processing, thus leading to slower responses for extrinsic grouping cues.

Additionally, due to the great similarity between proximity and common region (proximity is distributed in four quadrants, while the common region is divided into four quadrants by the border), we compared the grouping effects of proximity and common region. As shown in Fig. 3A, for RTs, proximity has a grouping effect (the grouping condition reacts significantly faster than the no-grouping condition), while common region shows no grouping effect (the difference between the grouping condition and no-grouping is not significant). For CV, both proximity and common region have a strong grouping effect. The CV under the grouping condition is significantly lower than that under the no-grouping condition, and the grouping effect of the common region is stronger (the difference between the grouping condition and no-grouping condition is more significant, *p* < 0.001) than proximity. Future research should select preschool children or first-grade primary school children to explore whether children who have not studied mathematics or have no complete magnitude representation system have different groupitizing effects given different grouping cues.

In recent years, significant progress has been made in the visual science of perceptual grouping. Recent studies have focused on the temporal processes and neural basis of intrinsic and extrinsic perceptual grouping^26,39^. For intrinsic grouping cues, grouping by proximity was found to be related to the positive component at the occipital electrode, whose amplitude peaks 100 to 120 ms after stimulus onset. The collinearity contour integral emerged 130 ms after stimulation^40^. Grouping by similarity (shape or color) appeared much later, and after 300 ms from stimulus onset, the negative occipito-temporal wave was activated^22,39^. In contrast, the neural basis of extrinsic grouping principles has received less attention. Montoro et al. (2015) reported the neural effects associated with grouping by common regions. They found that common region grouping cues belong to the category of long-latency grouping principles, which primarily involve activity in extrastriate cortices^22,28^. Future research should continue to explore the time course and neural mechanisms underlying intrinsic and extrinsic grouping cues of grouping effects.

Interestingly, we found that RTs for grouping by shape similarity were significantly lower than those of the other groupings (Fig. 3). Studies have shown that, in the absence of other visual information, it is easy for human beings to identify objects by shape^41–43^. Adults and children prefer to categorize novel objects according to shapes, given conflicting colors and texture cues. Moreover, shape features play a more important role in inductive reasoning than do color features^42,43^. Shape similarity is the first strategy used in inductive reasoning in early childhood^44^. Researchers presented subjects with reference stimuli of color, shape, and texture (such as square, blue, and wooden). They then presented two test stimuli with different shapes, colors, and textures, so that children could judge whether the test stimulus was consistent with the reference stimulus^45^. The results showed that 2–3 year old children chose shapes as the basis of induction. Future studies should select developing children as participants to explore whether the grouping effect of quantity estimation in shape similarity is different between children and adults.

Regarding math knowledge, for RTs, the interaction between math knowledge and grouping condition was significant (Fig. 6A). The grouping conditions for the high math knowledge group differed significantly in RT, while the middle and low math knowledge groups did not exhibit such a difference, indicating that the groupitizing effect benefited the most with high math knowledge. Many studies have demonstrated that an efficient ANS may be a prerequisite for the typical development of math skills^10,11^. Therefore, we speculate that the high math knowledge group had a more refined ANS, and that the groupitizing strategy was automatically used in quantity estimation. In the grouping condition, items were visually divided into subgroups, and since individuals with high levels of math knowledge could make better use of groupitizing strategy, RTs in the grouping condition were significantly faster than those in the no-grouping condition. Although sensory precision was higher in the grouping condition for the middle and low math knowledge groups, RTs did not differ among conditions. This may be because, in the grouping condition, when they used groupitizing strategies, they needed to employ more cognitive resources and required more time. In the no-grouping condition, they could not use any strategies; they could only make rough guesses based on their feelings. Thus, RTs were faster but precision was lower. However, for CV, the interaction between math knowledge and grouping condition was not significant (Fig. 6B); this means that high, medium, and low math knowledge groups all have strong grouping effects, which shows that each group benefits from groupitizing in numerosity perception; It further proves that groupitizing is an effective strategy in numerosity perception.

The present study found that, in the grouping condition, numbers 6, 9, 12, and 16 were associated with faster RTs and lower coefficients of variation than adjacent numbers. This may be because those specific numerosities’ configurations (6: 3, 3 = 2 × 3 = 2 groups of 3, 9: 3, 3, 3 = 3 × 3 = 3 groups of 3, 12: 4, 4, 4 = 3 × 4 = 3 groups of 4, 16: 4, 4, 4, 4 = 4 × 4 = 4 groups of 4) were divided into “equal groups,” which let the subjects use mental multiplication in their estimations. This is similar to the result of Dehaene et al. (2020)^4^, who found that for 5, 7, 11, and other such prime numbers, RTs were slower than for their neighbors, and for non-prime numbers, which could be subdivided into equal numbers, RTs were faster than for their neighbors (Fig. 5).

## Conclusion

The present study demonstrates that visually dividing an array into subgroups promotes numerosity perception. Moreover, our research combined the groupitizing effect of numerosity estimation with Gestalt theory for the first time. It also demonstrated a difference between the groupitizing effect of extrinsic versus intrinsic grouping cues, based on Palmer et al.^23–25^. The results thus suggest that it takes longer for to estimate numerosities given extrinsic grouping cues. However, the precision of extrinsic grouping cues is higher than that of intrinsic grouping cues due to a stronger groupitizing effect.

### Limitations and future directions

Since the concept of “groupitizing” was proposed by Wender and Roth Kegel (2000)^17^ and Starkey and McCandless (2014)^13^, studies have continued to explore the effect of grouping. This study combined the grouping effect and perceptual grouping principles, thus extending the study of groupitizing and the field of perceptual grouping.

Recent studies have begun to explore the shared associative mechanisms between different perceptual features. For example, the theory of magnitude model^46,47^ proposes that the parietal cortex of human beings processes quantitative information about space, time, and numbers together to optimize action plans and execution. It is necessary to explore the relationship between magnitude representation, space, and time. In this study, we only studied the grouping effect in space. However, subsequent studies should verify the differences in the grouping effect between intrinsic and extrinsic grouping cues in the time dimension.

The participants in this study were adults. Although they were divided into high, middle, and low math knowledge groups, the difference in the math level of adults is not very prominent. Many studies have found that in the process of development and formal arithmetic learning, numerosity perception precision has been greatly improved. In contrast, symbolic mathematics abilities in educated adults may have been stably mapped into their basic non-symbolic representation, making this connection less obvious^46–48^. Future research should explore the differences in groupitizing effects between preschool children with a low number sense and adults, as well as between children with difficulties in math and children without math difficulties.

To date, there has been no electrophysiological or neuroscience study that explores the grouping strategies between intrinsic and extrinsic cues. Future research should be conducted from the perspective of electrophysiology to investigate the neurofunctional links between grouping strategies and intrinsic and extrinsic cues, which would delineate a possible neural hierarchical model for “groupitizing.”

## Data Availability

Materials and data are available on our OSF page: https://osf.io/m5ay7/.
